# Chronopharmacological effects of 5-aminosalicylic acid in the treatment of experimental colitis in mice

**DOI:** 10.3389/fimmu.2026.1936952

**Published:** 2026-10-02

**Authors:** Yulin Wen, Shuangye Li, Jincan Jian, Jiahui Liu, Shuai Wang, Haomeng Wu

**Affiliations:** Chinese Medicine Guangdong Laboratory (Hengqin), State Key Laboratory of Traditional Chinese Medicine Syndrome, Key Laboratory of Chinese Medicinal Resource from Lingnan, The Second Affiliated Hospital of Guangzhou University of Chinese Medicine, Guangzhou University of Chinese Medicine, Guangzhou, China

**Keywords:** 5-aminosalicylic acid, experimental colitis, circadian clock, BMAL1, FTH1

## Abstract

**Background:**

5-Aminosalicylic acid (5-ASA) is a first-line therapy for ulcerative colitis, but whether dosing timing influences its therapeutic efficacy remains unclear. This study aims to investigate the chronopharmacological effects of 5-ASA in experimental colitis.

**Methods:**

A dextran sulfate sodium (DSS)-induced colitis model was used to compare the therapeutic effects of 5-ASA administered at different Zeitgeber times. Colonic proteomic profiling was performed to identify circadian-responsive proteins associated with differential drug efficacy. Intestinal epithelial-specific *Bmal1* knockout mice were used to determine whether the circadian clock regulates the chronopharmacological response to 5-ASA.

**Results:**

5-ASA showed marked time-dependent efficacy in DSS-induced colitis, with greater protection at ZT14 than at ZT2. Proteomic analysis identified ferritin heavy chain 1 (FTH1) as a circadian-responsive protein associated with differential treatment outcomes. 5-ASA suppressed FTH1 expression, whereas FTH1 overexpression attenuated anti-inflammatory effects of 5-ASA. Moreover, intestinal epithelial-specific deletion of *Bmal1* largely abolished diurnal variation in both FTH1 expression and 5-ASA efficacy, indicating that the intestinal clock coordinates the time-dependent response to 5-ASA.

**Conclusion:**

These findings identify FTH1 as a circadian-responsive factor linked to the time-dependent efficacy of 5-ASA and support circadian-guided optimization of 5-ASA administration as a potential strategy for improving UC treatment.

## 1 Introduction

Chronotherapy aims to optimize therapeutic efficacy and reduce adverse effects by aligning drug administration with endogenous circadian rhythms (1). Clinically, chronotherapy has demonstrated therapeutic benefits across multiple disease settings, including chronomodulated chemotherapy and time-adjusted administration of antihypertensive and lipid-lowering agents (2, 3). Both pharmacokinetic and pharmacodynamic processes are under circadian regulation, resulting in pronounced time-of-day–dependent variability in drug responses. Circadian clocks orchestrate key determinants of drug disposition, including absorption, distribution, metabolism, and excretion, while also regulating the rhythmic activity of pharmacological targets and downstream biological processes such as inflammation, oxidative stress, and cell death pathways (4, 5). These findings highlight dosing time as an important and clinically actionable determinant of drug efficacy and safety.

Ulcerative colitis (UC) is a chronic relapsing inflammatory disorder of the colon characterized by persistent mucosal inflammation and progressive epithelial injury (6). Despite advances in therapeutic strategies, including aminosalicylates, corticosteroids, immunosuppressants, biologics, and small-molecule agents, a substantial proportion of patients fail to achieve sustained remission or experience frequent disease relapse (7–11). These limitations highlight the substantial unmet clinical need for strategies that improve therapeutic response and long-term disease control. In addition, disease heterogeneity and interindividual variability in treatment response further complicate clinical management, underscoring the importance of optimizing existing therapeutic approaches. 5-Aminosalicylic acid (5-ASA), also known as mesalazine, remains the first-line therapy for patients with mild to moderate UC and is widely used for both induction and maintenance of remission (12, 13). Its therapeutic effects are primarily localized to the colonic mucosa, where it exerts anti-inflammatory activity through suppression of pro-inflammatory signaling pathways, attenuation of oxidative stress, and preservation of epithelial barrier integrity (14–18). These coordinated actions contribute to mucosal healing and disease control in UC (19). However, despite its well-established clinical efficacy, patient responses to 5-ASA are highly variable, and the determinants of this variability remain incompletely understood. In particular, whether dosing timing contributes to differential therapeutic outcomes of 5-ASA has not been systematically investigated.

In this study, we investigated the chronopharmacological effects of 5-ASA in experimental colitis and explored the role of the intestinal circadian clock in regulating its therapeutic efficacy. We identified zeitgeber time 14 (ZT14) as the optimal dosing time for maximal anti-inflammatory efficacy. Moreover, intestinal BMAL1-dependent circadian regulation coordinates time-dependent expression of FTH1, thereby gating the therapeutic response to 5-ASA. These results establish dosing timing as a critical determinant of 5-ASA efficacy and provide a rationale for chronotherapeutic optimization in UC.

## 2 Materials and methods

### 2.1 Animals

Intestinal epithelial-specific *Bmal1* knockout (IKO) mice were generated by crossing FLOX mice (The Jackson Laboratory, Bar Harbor, ME, USA) with *Villin*-Cre mice. Wild-type C57BL/6 mice (8 weeks old, 22 ± 2 g body weight) were obtained from Guangzhou Ruige Biological Technology Co., Ltd, Guangzhou, China. Animals were maintained under specific pathogen-free (SPF) conditions in the animal facility of the School of Traditional Chinese Medicine, Guangzhou University of Chinese Medicine, under a controlled environment (22 °C ± 2 °C) with a 12-h light/dark cycle (lights on at 6:00 a.m., defined as zeitgeber time 0, ZT0), and had *ad libitum* access to standard rodent chow and water. Mice were acclimatized to the housing conditions for at least 1 week before the experimental procedures.

All experimental procedures were performed in accordance with the Guidelines for the Care and Use of Experimental Animals and approved by the Animal Ethics and Use Committee of Guangzhou University of Chinese Medicine (ZYD-2026-061). As the experimental procedures were non-invasive, anesthesia was not administered prior to euthanasia. Mice were euthanized via carbon dioxide (CO_2_) inhalation at a controlled displacement rate (30%–70% volume/min), followed immediately by cervical dislocation to ensure death, in compliance with the AVMA Guidelines (2020). Death was confirmed by respiratory arrest and the absence of reflexes. All efforts were made to comply with the principles of the 3Rs to minimize animal suffering and ensure experimental reliability and reproducibility.

### 2.2 Generation of intestinal epithelial-specific *Bmal1* knockout mice

Intestinal epithelial-specific *Bmal1* knockout (IKO) mice on a C57BL/6J background were generated using the Cre–LoxP recombination system. Briefly, FLOX mice were crossed with *Villin*-Cre transgenic mice to obtain *Bmal1*^flox/flox^, *Villin*-Cre^+^ offspring, in which *Bmal1* was selectively deleted in intestinal epithelial cells. Littermate *Bmal1*^flox/flox^, *Villin*-Cre^−^ mice, carrying the same genetic background but lacking Cre recombinase, were used as control mice to ensure intact Bmal1 expression in all tissues.

Genomic DNA for genotyping was extracted from clipped and numbered tail tips using lysis buffer and proteinase K digestion, followed by ethanol precipitation and washing. PCR amplification was performed using specific primers (**Table 1**), and the resulting products were separated by agarose (Aladdin, China) gel electrophoresis. Mouse genotypes were determined based on the presence and size of specific DNA bands after gel imaging. Genotypes were assigned based on the presence of specific amplicons: 431 and 324 bp bands (FLOX, control) and 431, 324, and 567 bp bands (IKO).

**Table 1:** Sequences of specific primers.

Primer	Forward (5′ to 3′)	Reverse (5′ to 3′)
*Villin-Cre-1(mut)*	GTGTTTGGTTTGGTTTCCTCTGCATAAGA	GCAGGCAAATTTTGGTGTACGGTCA
*Villin-Cre-2(wild)*	CTAGGCCACAGAATTGAAAGATCT	GTAGGTGGAAATTCTAGCATCATCC
*Bmal1* ^flox/flox^	ACTGGAAGTAACTTTATCAAACTG	CTGACCAACTTGCTAACAATTA

### 2.3 Dextran sulfate sodium-induced colitis

Mice were randomly divided into 5-ASA and vehicle treatment groups, with six mice per group. The mice received 3% (w/v) dextran sulfate sodium (DSS; IVSCI, Shanghai) in drinking water *ad libitum* for 11 consecutive days (20). In the 5-ASA treatment groups, mice were simultaneously exposed to 3% DSS and received daily intragastric administration of 5-ASA (0.5 g/kg) at designated zeitgeber time points (ZT2, ZT6, ZT10, ZT14, ZT18, and ZT22) for the first 7 days, after which DSS exposure was replaced with regular drinking water while 5-ASA administration at the corresponding zeitgeber time points was continued for an additional 4 days (21). On day 11, all mice were euthanized and colon tissues were collected for subsequent histological and molecular analyses. The symptoms of colitis were quantified using the disease activity index (DAI) with minor modifications. Briefly, body weight loss was scored as 0 (0%), 1 (1%–5%), 2 (5%–10%), 3 (10%–20%), and 4 (>20%); stool consistency was scored as 0 (normal), 1 (soft stools), 2 (loose stools), and 3 (diarrhea); and rectal bleeding was scored as 0 (normal), 1 (mild bleeding in stool), 2 (moderate bleeding), and 3 (gross bleeding) (22, 23). Body weight, stool consistency, and fecal occult blood were monitored daily at 9:00 a.m., and each parameter was independently evaluated by two blinded investigators; the average score was used for analysis. The DAI was calculated as the mean value of the three subscores: DAI = (body weight loss score + stool consistency score + rectal bleeding score)/3.

### 2.4 Hematoxylin and eosin staining

Colon tissues were fixed in 4% paraformaldehyde for 12 h, followed by running water washing and gradient dehydration. Samples were sequentially cleared in a xylene–ethanol mixture and pure xylene for 1 h, then infiltrated three times with molten paraffin at 60 °C and embedded to form paraffin blocks. For section staining, slides were dewaxed in xylene and rehydrated via graded ethanol. Nuclei were stained with hematoxylin for 3–5 min, differentiated in 1% hydrochloric acid ethanol after water rinsing, and blued under running tap water. After graded ethanol dehydration, cytoplasm was stained with eosin alcohol solution for 5 min. Slides were further dehydrated and cleared with absolute ethanol and xylene, and mounted with neutral balsam for preservation.

### 2.5 Cell culture, transfection, and treatment

MODE-K cells (SSRCC, China) were cultured in high-glucose DMEM (Gibco, USA) supplemented with 10% fetal bovine serum (BidePharm, China) and 1% penicillin–streptomycin (Beyotime, China) at 37 °C in a humidified atmosphere containing 5% CO_2_. MODE-K cells were seeded in 6-well plates. When the cells have grown to an appropriate density, they were treated with 5-ASA (0, 50, and 100 µg/mL). At 24 h post-treatment, the optimal concentration of 5-ASA was determined based on Western blot quantification of FTH1 (Proteintech, USA) protein expression and used for subsequent experiments.

MODE-K cells were seeded in 6-well plates and transfected at appropriate confluence. The transfection plasmids include *Fth1* overexpression plasmid and corresponding negative control vector. Cells were transfected with 1 μg/mL plasmid per well using jetPRIME (Polyplus, France) transfection procedures. Successful establishment of the *Fth1* overexpression model was confirmed by quantitative real-time PCR (RT-qPCR) and western blot analysis. Similarly, the efficiency of *Fth1* knockdown was verified by RT-qPCR. To evaluate the effect of *Fth1* knockdown on the LPS-induced inflammatory response, cells were stimulated with LPS after transfection, and the mRNA expression levels of Tnf-α and Il-β were subsequently measured by RT-qPCR.

MODE-K cells were first transfected with *Fth1* overexpression plasmid (1 μg/mL) for 24 h. Following transfection, cells were stimulated with 2 μg/mL lipopolysaccharide (LPS, Sigma, USA) for 12 h to induce an inflammatory state. Subsequently, cells were treated with 5-ASA (100 μg/mL) for 24 h under continued experimental conditions, depending on the specific group design. Cells treated with equal volumes of vehicle served as solvent control. After treatment, total cellular protein and RNA were collected from each group. *Fth1* knockdown was achieved using siRNA transfection, and the siRNA sequences are provided in **Table 2**.

**Table 2:** siRNA sequences used for Fth1 knockdown in MODE-K cells.

Gene	Reagent	Sense (5′–3′)	Antisense (5′–3′)
Fth1	siRNA	GAACAGGUGAAAUCCAUUAAATT	UUUAAUGGAUUUCACCUGUUCTT

### 2.6 Enzyme-linked immunosorbent assay

The concentrations of IL-1β and TNF-α in MODE-K cell culture supernatants were measured using commercially available mouse enzyme-linked immunosorbent assay (ELISA) kits (Jianglai Biotechnology, China) according to the manufacturer’s instructions. Prior to analysis, culture supernatants were diluted fivefold with the appropriate sample diluent. Standards and diluted samples were added to the designated wells and sequentially incubated with the detection reagents and substrate solution provided with the kits. The enzymatic reaction was terminated with stop solution, and absorbance was measured at 450 nm using a microplate reader. Cytokine concentrations were calculated from the corresponding standard curves, corrected for the fivefold dilution factor, and expressed as ng/L.

### 2.7 Total RNA extraction and quantitative real-time PCR

Total RNA was extracted from mouse colon tissues using TRIzol reagent (Thermo Fisher, USA) according to the manufacturer’s instructions. Tissue samples were homogenized, and RNA was isolated through chloroform extraction and isopropanol (Macklin, China) precipitation, followed by washing with 75% ethanol and resuspension in RNase-free water. RNA concentration and purity were assessed prior to downstream processing.

Complementary DNA (cDNA) was synthesized using a reverse transcription kit (Vazyme, Nanjing, China). Quantitative real-time PCR was performed using an SYBR Green-based qPCR Master Mix (Vazyme, China) on a real-time PCR system. Relative gene expression levels were calculated using the 2−ΔΔCt method with Gapdh (Proteintech, USA) as the internal control (24). The primer sequences used are listed in **Table 3**.

**Table 3:** qPCR primer sequences.

Gene	Forward (5′ to 3′)	Reverse (5′ to 3′)
*Gapdh*	CAAGGAGTAAGAAACCCTGGA	CGAGTTGGGATAGGGCCTCT
*Tnf-α*	AGGGTCTGGGCCATAGAACT	CCACCACGCTCTTCTGTCTAC
*Il-1β*	AATGCCACCTTTTGACAGTGATG	AGCTTCTCCACAGCCACAAT
*Fth1*	CGAGGTGGCCGAATCTTCC	TGCAGTTCCAGTAGTGACTGAT

### 2.8 Proteomic analysis

Proteomic analysis was performed on colonic tissues collected after 11 days of drug administration. Tissue samples were washed with PBS and homogenized in lysis buffer to extract total proteins. Protein concentration was determined using a BCA assay (Beyotime, China), followed by quality control via SDS-PAGE. Proteins were denatured, reduced, and alkylated prior to enzymatic digestion with trypsin (Gibco, USA). The resulting peptides were desalted using C18 columns, dried, and reconstituted in 0.1% formic acid (GHTech, China) for LC-MS/MS analysis. Peptide separation was performed using a Vanquish Neo UHPLC system (Thermo Scientific, USA), and mass spectrometric acquisition was carried out on an Orbitrap Astral mass spectrometer (Thermo Scientific, USA) in data-independent acquisition (DIA) mode. Raw DIA data were processed using DIA-NN 1.9 software against a reference proteome database. A spectral library was generated using integrated deep learning-based algorithms combined with match-between-runs (MBR) for enhanced peptide identification. Protein identification was filtered at a 1% false discovery rate (FDR) at both peptide and protein levels, and the resulting quantitative proteomic data were used for downstream analysis.

### 2.9 Western blotting

Fresh colon tissues harvested on day 11 post-drug administration were weighed (~50 mg) and homogenized in RIPA lysis buffer (Beyotime, China) using grinding beads on ice. Cultured cells were directly lysed in RIPA buffer (Beyotime, China) under the same conditions. Lysates were centrifuged at 4 °C, and the supernatants were collected for protein extraction. Protein concentration was determined using a BCA assay (Beyotime, China), followed by normalization to equal loading amounts.

Samples were denatured by heating with 5× SDS loading buffer (NCM Biotech, China) containing protease inhibitor (MedChemExpress, USA) at a 1:5 ratio. Proteins were separated on 10% SDS-PAGE gels (Beyotime, China) and transferred onto PVDF membranes (Millipore, USA) using a wet transfer system. Membranes were blocked with skim milk at room temperature, followed by incubation with primary antibodies at 4 °C overnight and corresponding secondary antibodies (HRP-conjugated Goat Anti-Rabbit IgG(H+L)/HRP-conjugated Goat Anti-Mouse IgG(H+L), Proteintech, Cat No.SA00001-2/SA00001-1, China) at room temperature. After TBST washing, protein bands were visualized using enhanced chemiluminescence (ECL) reagent (Vazyme, China) and detected by chemiluminescence imaging.

The primary antibodies were as follows: GAPDH polyclonal antibody (Proteintech, 1:5,000, Cat. No.10494-1-AP), TNF-alpha monoclonal antibody (Proteintech,1:5,000, Cat. No. 60291-1-Ig), IL-1 beta polyclonal antibody (Proteintech, 1:1,000, Cat. No. 26048-1-AP), and ferritin heavy chain polyclonal antibody (Proteintech, 1:3,000, Cat. No. 11682-1-AP).

### 2.10 LC–MS-based pharmacokinetic analysis

Plasma samples were prepared from mice administered 5-ASA and analyzed by LC–MS. Briefly, 12 wild-type mice fasted for 12 h were orally gavaged with 5-ASA (0.5 g/kg body weight; Aidefa Pharmacy, Shanghai, China) at ZT2 or ZT14. Blood samples were collected 5, 20, 30, 60, and 120 min post-administration, and plasma was separated by centrifugation. Proteins were precipitated using acetonitrile (GHTech, China, plasma:ACN = 1:3, v/v), followed by vortexing and centrifugation at 4 °C. The supernatant was collected for LC–MS analysis.

Chromatographic separation was performed using an ACQUITY Premier UPLC system equipped with a BEH C18 column (2.1 × 50 mm, 1.7 μm). The mobile phase consisted of 0.1% formic acid (GHTech, China) in water (A) and methanol (Aladdin, China) (B) at a flow rate of 0.3 mL/min with the following gradient: 0–8 min, 5%–95% B; 8–8.5 min, 95%–5% B; 8.5–12 min, 5% B. Mass spectrometric detection was performed using a Xevo G3 QTof (Waters Corporation, Milford, MA, USA) in negative ESI–MSE mode (*m*/*z* 50–1,200). Key parameters included a capillary voltage of 2 kV, cone voltage of 45 V, collision energy of 20–30 eV, source temperature of 100 °C, and desolvation temperature of 250 °C (desolvation gas flow: 600 L/h; cone gas flow: 50 L/h).

### 2.11 Statistical analysis

All statistical analyses were performed using GraphPad Prism software. Data are presented as mean ± standard deviation (SD). For comparisons between two experimental groups, statistical significance was determined using two-tailed unpaired Student’s *t*-test. Comparisons among three or more groups were performed using one-way ANOVA followed by Sidak’s multiple-comparisons test. When two independent variables were involved, two-way ANOVA followed by Sidak’s multiple-comparisons test was used. Statistical significance was defined as *p* < 0.05. Graphical representations were generated using GraphPad Prism 9.5 software.

## 3 Results

### 3.1 5-ASA exhibits dosing time-dependent therapeutic efficacy in colitis

To determine whether the therapeutic efficacy of 5-ASA in colitis is dosing time-dependent, a DSS-induced colitis model was established and 5-ASA was administered at different zeitgeber times (**Figure 1A**). Time-of-day-dependent differences in therapeutic efficacy were observed across treatment groups. Administration at ZT14 produced the greatest therapeutic benefit, as evidenced by reduced DAI, attenuated body weight loss, and accelerated recovery compared with other time points (**Figures 1B, C**). Consistently, ZT14 treatment most effectively alleviated DSS-induced colon shortening and histopathological injury (**Figures 1D–G**), whereas ZT2 administration showed the weakest efficacy. Correspondingly, both mRNA and protein expression levels of pro-inflammatory cytokines were significantly lower in the ZT14 group than in the ZT2 group (**Figures 1H, I**). Collectively, these results demonstrate that the anti-inflammatory effects of 5-ASA in colitis are strongly associated with dosing timing, with ZT14 representing the optimal therapeutic window.

**Figure 1:**
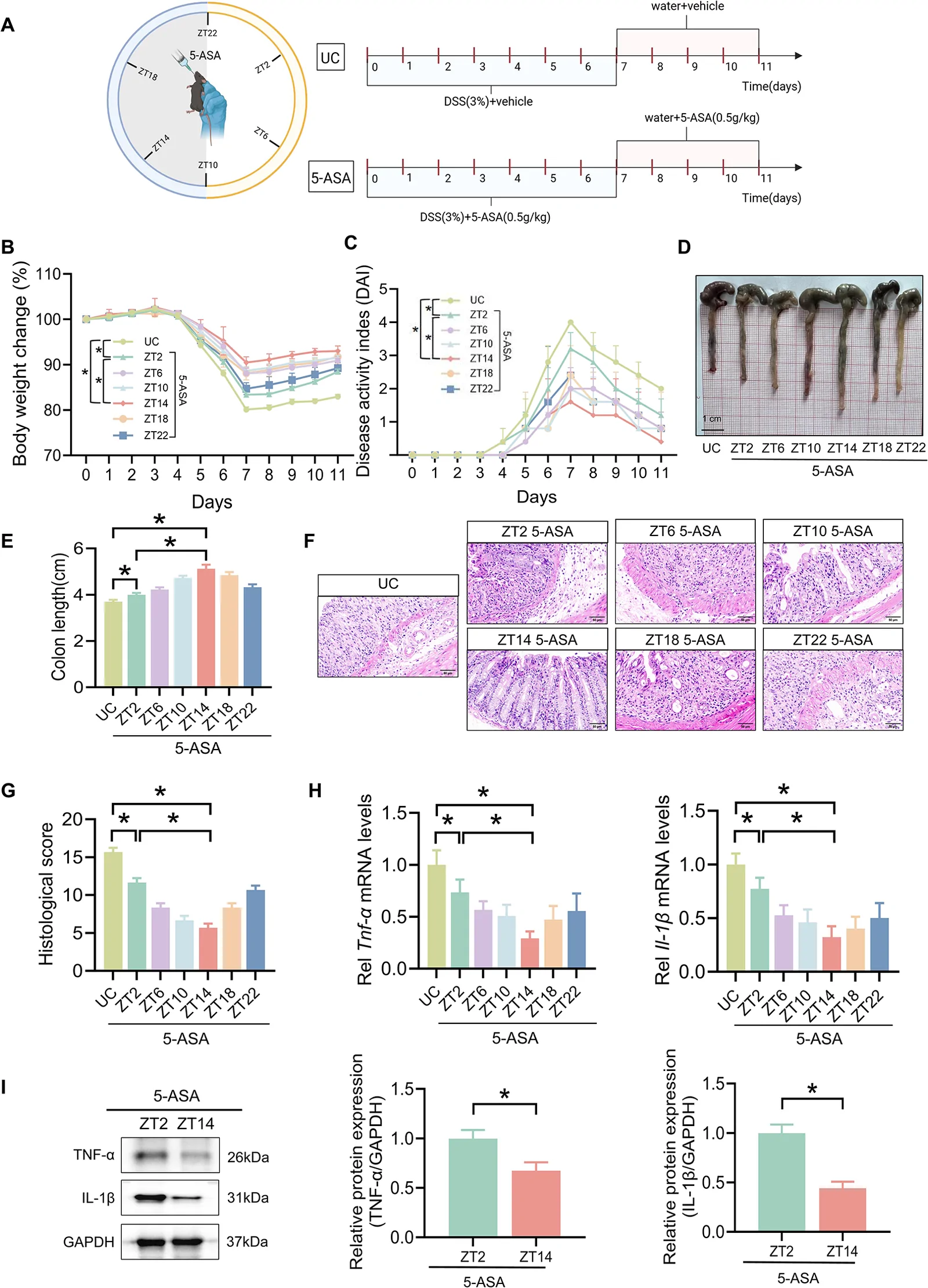
5-ASA exhibits dosing time-dependent therapeutic efficacy in colitis. **(A)** Schematic diagram of 5-ASA administration regimens at six time points. **(B)** Body weight changes of mice during the 7-day DSS modeling period and 4-day recovery period (*n* = 6). **(C)** Disease activity index (DAI) evaluation of colitis in mice (each treatment group vs. the UC group, *n* = 6). **(D)** The external morphology of colons derived from mice treated at different time points after modeling completion (*n* = 6) (representative images). **(E)** Colon length of mice treated with 5-ASA at different time points *(n* = 6). **(F)** H&E staining of colon derived from mice treated at different time points (*n* = 6). **(G)** Histopathological assessment of colon tissues from colitis mice. **(H)** Relative mRNA expression of *Tnf-α* and *Il-1β* in colon tissues determined by qPCR (each treatment group vs. the UC group, *n* = 6). **(I)** Western blot and quantitative analysis of TNF-α and IL-1β protein abundances in colon tissues from ZT2 and ZT14 administration groups (treatment groups vs. the UC group, *n* = 6). Data are expressed as mean ± SD. *p < 0.05. ZT, zeitgeber time.

### 3.2 Proteomic profiling identifies FTH1 as a circadian-responsive biomarker associated with 5-ASA efficacy

Circadian variation in pharmacokinetics has been implicated in time-dependent differences in drug efficacy (25). To determine whether the chronoefficacy of 5-ASA was associated with differences in systemic drug exposure, mice were administered 5-ASA at ZT2 or ZT14, and serum samples were collected at five predefined time points after dosing to determine the concentrations of 5-ASA (**Figure 2A**). The serum concentration–time profiles and major pharmacokinetic parameters were comparable between the ZT2 and ZT14 groups, with no significant between-group differences observed (**Figure 2B**; **Table 4**). Similarly, serum concentrations of the N-acetyl-5-ASA did not differ significantly between the ZT2 and ZT14 groups (**Supplementary Figure S1**). These findings suggest that the difference in therapeutic efficacy between the two dosing times is unlikely to be primarily explained by differences in systemic pharmacokinetics.

**Figure 2:**
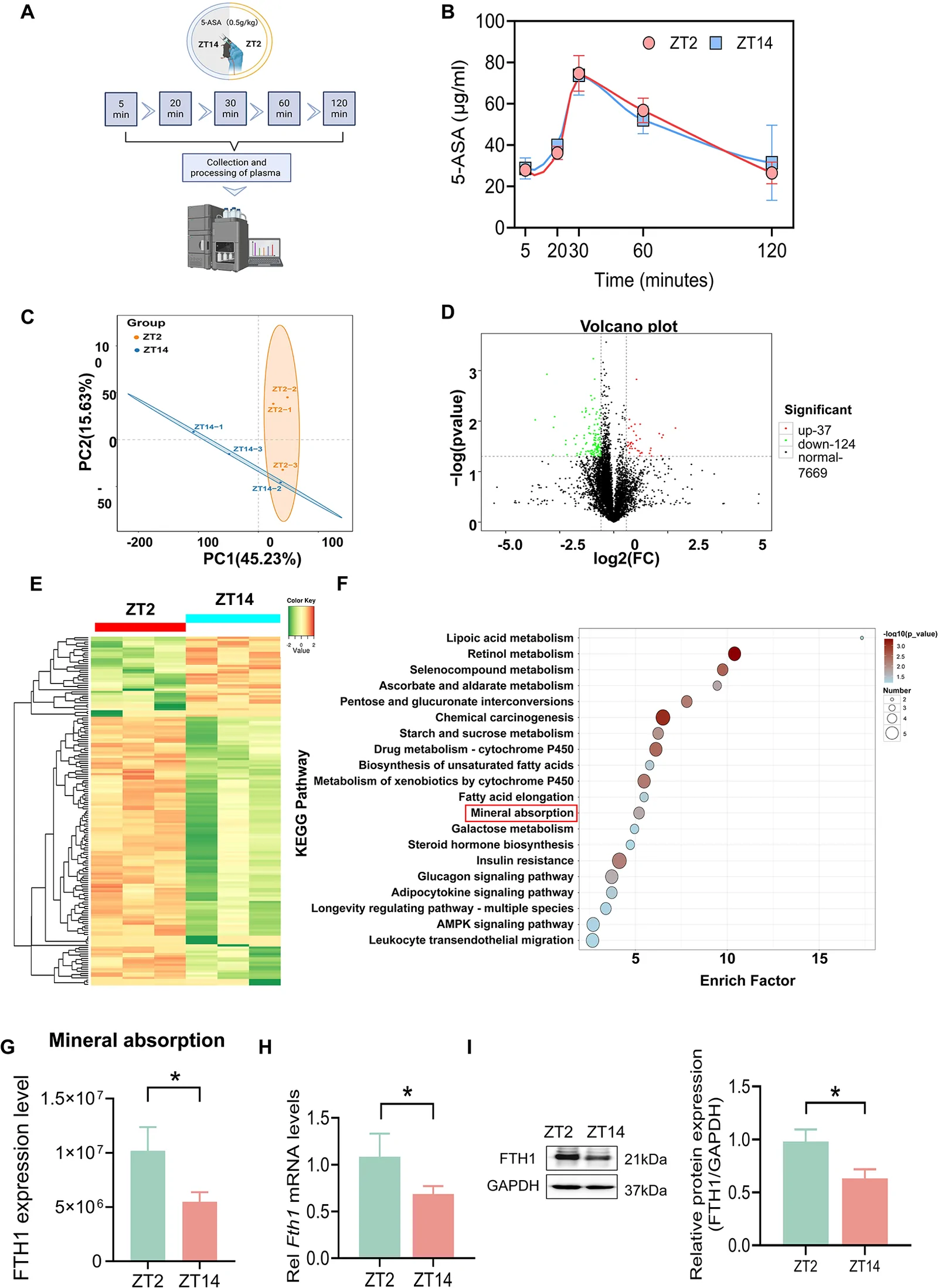
Proteomic profiling identifies FTH1 as a circadian-responsive target associated with 5-ASA efficacy. **(A)** Schematic diagram of mass spectrometric analysis to determine 5-ASA metabolic rate. **(B)** Plasma concentration–time profiles of 5-ASA following administration at ZT2 and ZT14. **(C)** PCA of proteomic analysis. **(D)** Volcano plot of differentially expressed proteins. **(E)** Heat map of differentially expressed proteins. **(F)** Bubble diagram of KEGG enrichment analysis. **(G)** Protein expression of FTH1 in the “mineral absorption” pathway (*n* = 3). **(H)** Comparison of mRNA expression of Fth1 in colon tissues from mice treated at ZT2 and ZT14 (*n* = 6). **(I)** Western blot detection and quantitative analysis of FTH1 protein in colons of mice administrated at ZT2 and ZT14 (*n* = 6). Data are expressed as mean ± SD (ns, not significant; *p < 0.05).

**Table 4:** Pharmacokinetic parameters of 5-ASA in mice following administration at ZT2 and ZT14.

Dosing time	*t*_1/2_ (min)	*T*_max_ (min)	*C*_max_ (μg/mL)	AUC_0–_*_t_* (μg/mL min)	AUC_0–inf_ (μg/mL min)	Vz/F (mg/kg)/(μg/mL)	Cl/F (mg/kg)/(μg/mL)/min
ZT2	59.386	30	74.706	5,578.384	7,851.497	5.456	0.06368
ZT14	74.284	30	73.773	5,551.620	8,920.486	6.007	0.05605

To explore potential biomarker, we performed quantitative proteomic profiling of colonic tissues from mice treated with 5-ASA at ZT2 or ZT14. Principal component analysis showed clear separation between the two groups (**Figure 2C**), indicating distinct proteomic landscapes associated with dosing time. Consistently, differential expression analysis revealed extensive remodeling of the colonic proteome between the two treatment conditions (**Figures 2D, E**). KEGG pathway enrichment analysis of the differentially expressed proteins identified mineral absorption as the most significantly altered pathway (**Figure 2F**). Within this pathway, ferritin heavy chain 1 (FTH1) was differentially expressed, with higher abundance in the ZT2 group than in the ZT14 group. This difference was further validated by qPCR and immunoblotting (**Figures 2G–I**). Collectively, these findings indicate that FTH1 expression varies according to the timing of 5-ASA administration and is associated with the time-dependent therapeutic response to 5-ASA, suggesting that FTH1 may serve as a biomarker of treatment response.

### 3.3 FTH1 is associated with the anti-inflammatory effects of 5-ASA

To investigate the role of FTH1 in inflammation, FTH1 was knocked down in MODE-K cells (**Figure 3A**). FTH1 knockdown significantly reduced the expression of pro-inflammatory cytokines, suggesting that FTH1 may promote inflammatory responses in intestinal epithelial cells (**Figure 3B**). Notably, 5-ASA reduced endogenous FTH1 protein expression in a dose-dependent manner (**Figure 3C**). To further determine whether FTH1 contributes to the anti-inflammatory effects of 5-ASA, we generated FTH1-overexpressing MODE-K cells and confirmed successful FTH1 overexpression at the protein level (**Figure 3D**). Treatment with 5-ASA significantly suppressed LPS-induced FTH1 upregulation and the production of inflammatory cytokines (**Figures 3E–G**). Notably, enforced FTH1 expression largely attenuated the inhibitory effect of 5-ASA on FTH1 protein abundance and concomitantly restored the levels of TNF-α and IL-1β that were suppressed by 5-ASA treatment (**Figures 3E–G**). Collectively, these findings suggest that FTH1 contributes to intestinal inflammatory responses and is associated with the anti-inflammatory activity of 5-ASA.

**Figure 3:**
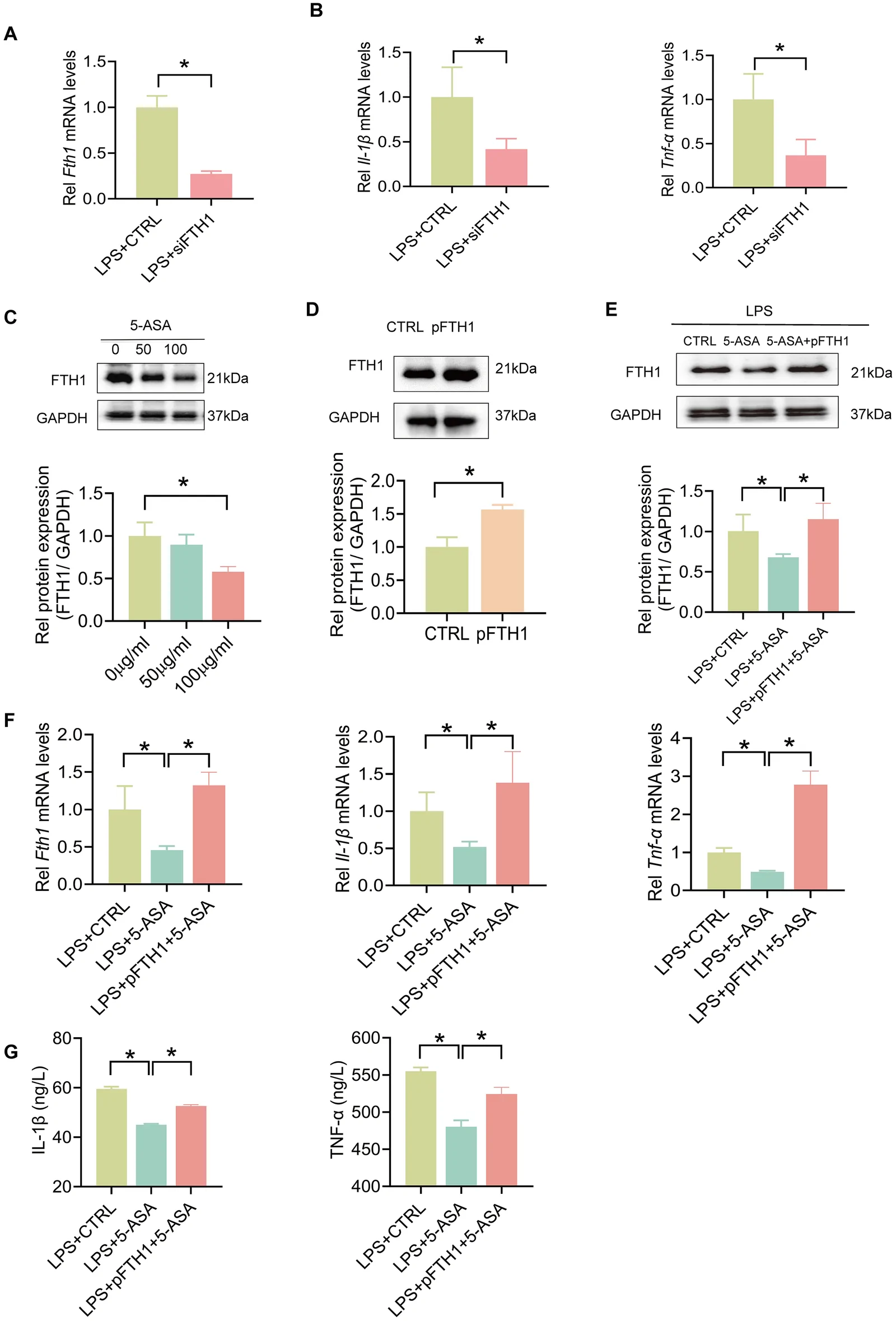
FTH1 mediates the anti-inflammatory effects of 5-ASA. **(A)** Relative mRNA expression of *Fth1* in MODE-K cells of LPS+Ctrl and LPS+siFTH1 groups under LPS stimulation (*n* = 3). **(B)** Relative mRNA expression of Il-1β *and Tnf-α* in MODE-K cells of LPS+Ctrl and LPS+siFTH1 groups under LPS stimulation (*n* = 3). **(C)** Western blot and quantitative analysis of FTH1 protein level in MODE-K cells treated with 0, 50, or 100 μg/mL 5-ASA (*n* = 3). **(D)** Western blot and quantitative analysis of FTH1 protein level in MODE-K cells from the Ctrl and pFTH1 groups (*n* = 3). **(E)** Western blot and quantitative analysis of FTH1 protein level in MODE-K cells of LPS+Ctrl, LPS + 5-ASA, and LPS+pFTH1 + 5-ASA groups under LPS stimulation (*n* = 3). **(F)** Relative mRNA expression of *Fth1*, Il-1β, and *Tnf-α* in MODE-K cells of LPS+Ctrl, LPS + 5-ASA, and LPS+pFTH1 + 5-ASA groups under LPS stimulation (*n* = 3). **(G)** ELISA analysis of IL-1β and TNF-α levels in the culture supernatants of MODE-K cells from the LPS+Ctrl, LPS+5-ASA, and LPS+pFTH1 + 5-ASA groups (*n* = 3). Data are expressed as mean ± SD (*p < 0.05).

### 3.4 BMAL1-dependent circadian control is associated with the therapeutic timing of 5-ASA

BMAL1 is a core component of the molecular circadian clock, and its disruption leads to impaired circadian regulation (26). To investigate whether intestinal circadian signaling contributes to the time-dependent efficacy of 5-ASA, we generated intestinal epithelial-specific *Bmal1* knockout (IKO) mice (**Figure 4A**). Deletion of *Bmal1* was verified by PCR-based genotyping (**Figure 4B**). Expression of clock genes, including *Nr1d1*, *Nr1d2*, *Per2*, and *Npas2*, was significantly disrupted in IKO mice (**Figure 4C**). Importantly, intestinal *Bmal1* deletion abolished the temporal variation in *Fth1* expression observed in control mice, resulting in comparable levels at ZT2 and ZT14 (**Figure 4D**). We next assessed whether disruption of the circadian clock affects the chronotherapeutic response to 5-ASA. In FLOX mice, 5-ASA administered at ZT14 conferred greater protection against DSS-induced colitis than at ZT2, as reflected by improved body weight recovery and reduced colonic injury (**Figures 4E–G**). This time-dependent therapeutic difference was absent in IKO mice, which showed comparable disease severity regardless of administration time. Furthermore, TNF-α and FTH1 exhibited lower expression at ZT14 than at ZT2 in control mice, whereas this rhythmic pattern was lost following *Bmal1* deletion (**Figures 4H, I**). Collectively, these findings identify intestinal BMAL1 as a key regulator of the dosing time-dependent efficacy of 5-ASA.

**Figure 4:**
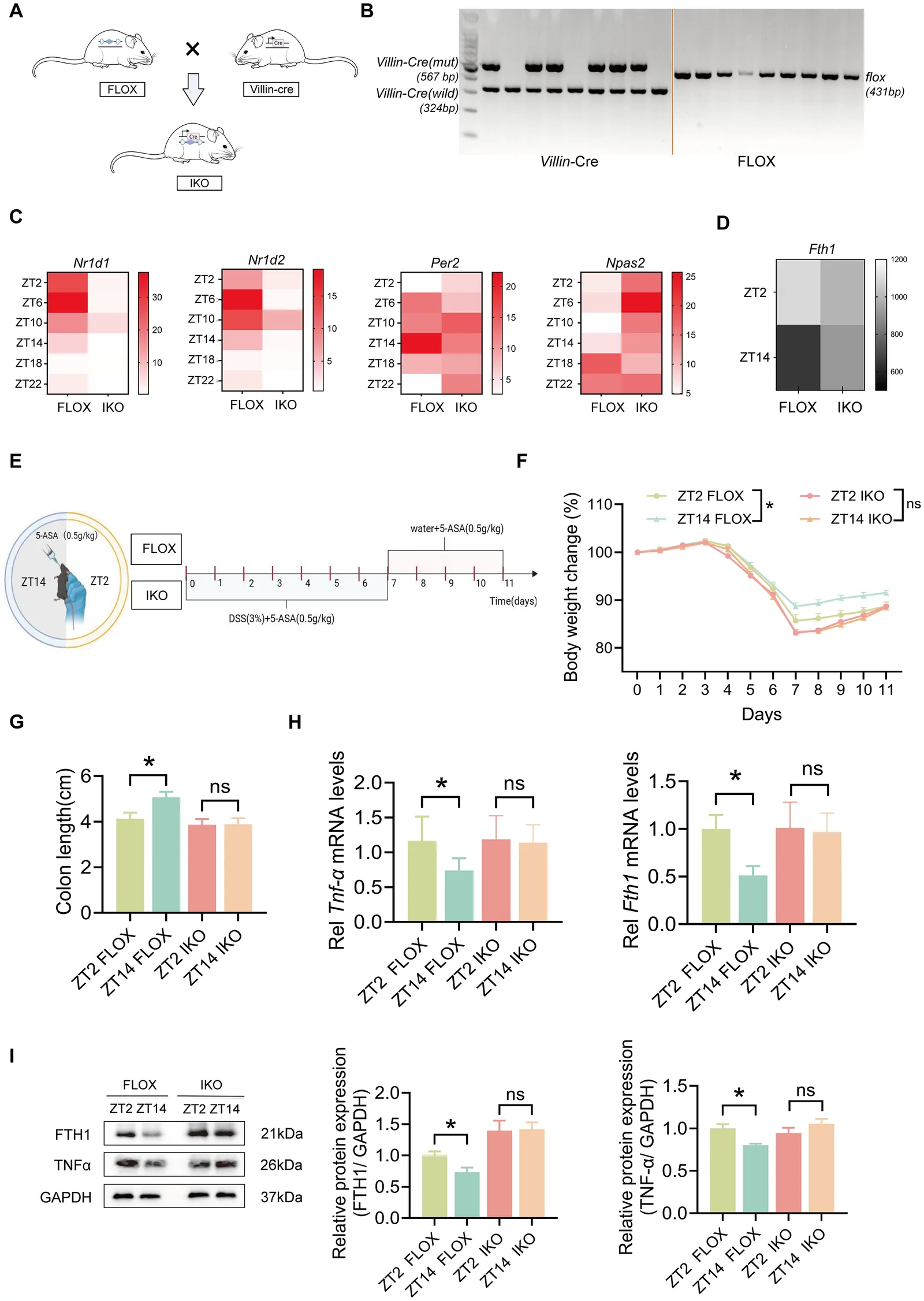
BMAL1-dependent circadian control is associated with the therapeutic timing of 5-ASA. **(A)** Construction methods of intestinal-specific *Bmal1* knockout (IKO) mice. **(B)** Genotype identification of IKO mice. **(C)** Heat map of core clock gene (*Nr1d1*, *Nr1d2*, *Per2*, and *Npas2*) expression across time points in FLOX and IKO mice. **(D)** Heat map of *Fth1* expression at ZT2 and ZT14 in FLOX mice and IKO mice. **(E)** Schematic diagram of 5-ASA administration procedures in FLOX and IKO mice at ZT2 and ZT14. **(F)** Body weight changes of IKO and FLOX mice treated with 5-ASA at ZT2 and ZT14 (ZT2 FLOX, ZT14 FLOX, ZT2 IKO, and ZT14 IKO, *n* = 6). **(G)** Colon length of IKO and FLOX mice treated with 5-ASA at ZT2 and ZT14 (*n* = 6). **(H)** Relative mRNA expression of *Tnf-α* and *Fth1* in colons of IKO and FLOX mice treated with 5-ASA at ZT2 and ZT14 (*n* = 6). **(I)** Western blot detection and quantitative analysis of FTH1 and TNF-α protein levels in colons of IKO and FLOX mice treated with 5-ASA at ZT2 and ZT14 (*n* = 6). Data are expressed as mean ± SD (ns, not significant; *p < 0.05).

## 4 Discussion

In this study, we demonstrate that 5-ASA exhibits chronopharmacological effects in DSS-induced colitis. The severity of DSS-induced colitis did not differ significantly among the different circadian time points under the same experimental conditions (27), supporting the interpretation that the observed differences primarily reflect time-of-day-dependent variation in 5-ASA efficacy. To achieve the primary objective of comparing treatment efficacy across multiple dosing times while minimizing animal use, a single time-matched control group was used rather than establishing a separate control group for each dosing time point. Administration at ZT14 resulted in superior therapeutic efficacy, as reflected by improved clinical and histological outcomes. Comparison of 5-ASA efficacy across different administration times is a key feature of chronopharmacological studies (28, 29). Dosing time-dependent response was accompanied by rhythmic expression of FTH1 in the intestine. Furthermore, intestinal epithelial-specific deletion of *Bmal1* abolished the temporal differences in 5-ASA efficacy, indicating that the intestinal circadian clock coordinates the chronopharmacological response to 5-ASA. Together, these findings identify dosing time as an important determinant of 5-ASA efficacy and support a role for circadian regulation in shaping therapeutic responses during intestinal inflammation.

FTH1 functions as a key regulator of intracellular iron homeostasis by sequestering redox-active iron and limiting ferroptotic cell death (30). Consistent with this role, impaired ferritin buffering has been associated with increased oxidative stress and exacerbated intestinal inflammation (31–33). Notably, we identified circadian oscillations in colonic FTH1 expression, indicating that iron-storage capacity may be under temporal regulation. While the present study does not establish a causal link between rhythmic FTH1 expression and ferroptosis, these observations support a model in which circadian control of iron metabolism may gate ferroptosis susceptibility across the day. Given the emerging role of ferroptosis in epithelial injury, temporal variation in ferroptotic sensitivity may represent an unrecognized mechanism contributing to daily fluctuations in intestinal inflammation and treatment responsiveness. Defining how circadian clocks coordinate iron metabolism and ferroptosis will therefore be an important direction for future investigation.

Emerging evidence has implicated BMAL1 in the regulation of iron metabolism, including pathways governing iron storage and antioxidant defense (34). Consistent with this concept, we found that intestinal epithelial-specific deletion of *Bmal1* abolished the rhythmic expression of FTH1, suggesting that BMAL1 contributes to the temporal regulation of FTH1. Notably, 5-ASA increased BMAL1-dependent luciferase activity (**Supplementary Figure S2**), indicating that circadian clock components are responsive to 5-ASA intervention. Integrating these findings, we propose a model in which 5-ASA partially exerts its chronopharmacological effects through BMAL1-dependent pathways. Because BMAL1 activity varies across circadian time, the ability of 5-ASA to modulate downstream targets such as FTH1 may likewise differ according to the timing of administration. Although the direct regulatory effects between BMAL1 and FTH1 remains to be established, our findings support a functional link between *Bmal1* regulation, FTH1 expression, and the dosing time-dependent therapeutic response to 5-ASA.

Our findings identify dosing timing as an important determinant of 5-ASA efficacy in colitis. Although the optimal dosing phase identified in mice cannot be directly translated into a specific clock time in humans because of interspecies differences in circadian characteristics, including differences in activity–rest cycles, our findings suggest that dosing phase may influence 5-ASA efficacy and provide a preclinical rationale for further evaluating time-dependent 5-ASA treatment in patients with UC. Moreover, the present study was conducted in an acute DSS-induced colitis model, whereas human UC is a chronic and relapsing disease. Whether the temporal efficacy profile observed here persists under chronic disease conditions, and whether it translates into improved therapeutic efficacy or long-term disease control in patients with UC, remains to be established. Furthermore, the present study also did not model different degrees of disease severity, and whether the time-dependent efficacy of 5-ASA is preserved under more severe inflammatory conditions remains to be established. Further studies using models with different degrees of disease severity will be required to determine whether and to what extent the time-dependent efficacy of 5-ASA is preserved under more severe inflammatory conditions. Additionally, the functional role of FTH1 in 5-ASA efficacy was demonstrated only *in vitro* and has not been validated *in vivo*.

In conclusion, this study demonstrates that 5-ASA exhibits chronopharmacological effects in experimental colitis, with superior therapeutic efficacy observed at ZT14. Our findings support a role for the intestinal clock in coordinating the dosing time-dependent response to 5-ASA. These findings expand our understanding of the circadian regulation of drug responses in intestinal inflammation and provide a conceptual framework for optimizing the timing of 5-ASA administration in the treatment of UC.

## Data Availability

The datasets presented in this study can be found in online repositories. The names of the repository/repositories and accession number(s) can be found below: PXD084486 (https://www.ebi.ac.uk/pride/archive/projects/PXD084486).

## References

[1] Cardinali DP Brown GM Pandi-Perumal SR . “Chronotherapy”. In: DF Swaab F Kreier PJ Lucassen A Salehi RM Buijs , editors. Handbook of Clinical Neurology. Vol. 179, The Human Hypothalamus: Anterior Region. Amsterdam: Elsevier (2021). p. 357–70. doi: 10.1016/B978-0-12-819975-6.00023-6 34225975

[2] Giacchetti S Bjarnason G Garufi C Genet D Iacobelli S Tampellini M . Phase III trial comparing 4-day chronomodulated therapy versus 2-day conventional delivery of fluorouracil, leucovorin, and oxaliplatin as first-line chemotherapy of metastatic colorectal cancer: The European Organisation for Research and Treatment of Cancer Chronotherapy Group. J Clin Oncol. (2006) 24:3562–9. doi: 10.1200/JCO.2006.06.1440 16877722

[3] Kaşkal M Sevim M Ülker G Keleş C Bebitoğlu BT . The clinical impact of chronopharmacology on current medicine. Naunyn Schmiedebergs Arch Pharmacol. (2025) 398:6179–91. doi: 10.1007/s00210-025-03788-7 39792169 PMC12125046

[4] Dallmann R Brown SA Gachon F . Chronopharmacology: New insights and therapeutic implications. Annu Rev Pharmacol Toxicol. (2014) 54. doi: 10.1146/annurev-pharmtox-011613-135923 24160700 PMC3885389

[5] Ayyar VS Sukumaran S . Circadian rhythms: Influence on physiology, pharmacology, and therapeutic interventions. J Pharmacokinet Pharmacodyn. (2021) 48:321–38. doi: 10.1007/s10928-021-09751-2 33797011 PMC8015932

[6] Krugliak Cleveland N Torres J Rubin DT . What does disease progression look like in ulcerative colitis, and how might it be prevented? Gastroenterology. (2022) 162:1396–408. doi: 10.1053/j.gastro.2022.01.023 35101421

[7] Singh S Loftus EV Limketkai BN Haydek JP Agrawal M Scott FI . AGA living clinical practice guideline on pharmacological management of moderate-to-severe ulcerative colitis. Gastroenterology. (2024) 167:1307–43. doi: 10.1053/j.gastro.2024.10.001 39572132 PMC12162142

[8] Wang Y Parker CE Feagan BG MacDonald JK . Oral 5-aminosalicylic acid for maintenance of remission in ulcerative colitis. Cochrane Database Syst Rev. (2016) 5:CD000544. doi: 10.1002/14651858.CD000544.pub4 27158764 PMC7045447

[9] Ribaldone DG Valdiserra G Di Salvo C Puxeddu I Bertani L Antonioli L . The right drug at the right time: Key to IBD success. Trends Mol Med. (2025) 32:658–77. doi: 10.1016/j.molmed.2025.10.010 41314864

[10] Pellegrino R Imperio G De Costanzo I Izzo M Landa F Tambaro A . Small molecules in the treatment of acute severe ulcerative colitis: A review of current evidence. Pharmaceuticals. (2025) 18:308. doi: 10.3390/ph18030308 40143087 PMC11944803

[11] Appiah JK Hayat U Garg N Asante R Donneyong E Haider MU . Emerging therapies in inflammatory bowel disease: A comprehensive review. J Clin Med. (2025) 14:6119. doi: 10.3390/jcm14176119 40943878 PMC12428888

[12] López-Alarcón C Rocco C Lissi E Carrasco C Squella JA Nuñez-Vergara L . Reaction of 5-aminosalicylic acid with peroxyl radicals: Protection and recovery by ascorbic acid and amino acids. Pharm Res. (2005) 22:1642–8. doi: 10.1007/s11095-005-6948-y 16180121

[13] Ye B van Langenberg DR . Mesalazine preparations for the treatment of ulcerative colitis: Are all created equal? World J Gastrointest Pharmacol Ther. (2015) 6:137–44. doi: 10.4292/wjgpt.v6.i4.137 26558148 PMC4635154

[14] Cevallos SA Lee J-Y Velazquez EM Foegeding NJ Shelton CD Tiffany CR . 5-aminosalicylic acid ameliorates colitis and checks dysbiotic Escherichia coli expansion by activating PPAR-γ signaling in the intestinal epithelium. mBio. (2021) 12:e03227-20. doi: 10.1128/mBio.03227-20 33468700 PMC7845635

[15] Ikehata A Hiwatashi N Kinouchi Y Ito K Yamazaki H Toyota T . 5-aminosalicylic acid inhibits leukotriene B4ω-hydroxylase activity in human polymorphonuclear leukocytes. Prostaglandins Leukot Essent Fatty Acids. (1994) 50:317–20. doi: 10.1016/0952-3278(94)90240-2 7938083

[16] Mbodji K Charpentier C Guérin C Querec C Bole-Feysot C Aziz M . Adjunct therapy of n-3 fatty acids to 5-ASA ameliorates inflammatory score and decreases NF-κB in rats with TNBS-induced colitis. J Nutr Biochem. (2013) 24:700–5. doi: 10.1016/j.jnutbio.2012.03.022 22841543

[17] Hayashi Y Aoyagi K Morita I Yamamoto C Sakisaka S . Oral administration of mesalazine protects against mucosal injury and permeation in dextran sulfate sodium-induced colitis in rats. Scand J Gastroenterol. (2009) 44:1323–31. doi: 10.3109/00365520903262414 19891583

[18] Kang S Kim W Jeong S Lee Y Nam J Lee S . Oxidized 5-aminosalicylic acid activates Nrf2-HO-1 pathway by covalently binding to Keap1: Implication in anti-inflammatory actions of 5-aminosalicylic acid. Free Radic Biol Med. (2017) 108:715–24. doi: 10.1016/j.freeradbiomed.2017.04.366 28473247

[19] Sonu I Lin MV Blonski W Lichtenstein GR . Clinical pharmacology of 5-ASA compounds in inflammatory bowel disease. Gastroenterol Clin North Am. (2010) 39:559–99. doi: 10.1016/j.gtc.2010.08.011 20951918

[20] Wu F Wang Y Mai Z Xu Z Li S Li Y . Human intestinal fungus Clavispora lusitaniae attenuates colitis through pyruvate decarboxylase-derived indole-3-ethanol. Nat Commun. (2025) 16:9980. doi: 10.1038/s41467-025-64914-w 41233306 PMC12615640

[21] Pan Z Liu J Lin S Li Z Qin S Wu H . Ji-ming-san enhances intestinal circadian rhythms and mitigates colitis in mice: The role of epithelial RORα. Phytomedicine. (2025) 142:156759. doi: 10.1016/j.phymed.2025.156759 40252431

[22] Jia L Jiang Y Wu L Fu J Du J Luo Z . Porphyromonas gingivalis aggravates colitis via a gut microbiota-linoleic acid metabolism-Th17/treg cell balance axis. Nat Commun. (2024) 15:1617. doi: 10.1038/s41467-024-45473-y 38388542 PMC10883948

[23] Li X Jin R Wang Z Niu C Song Z Liu X . TSP50 deficiency in neural stem cells aggravates colitis in mice by altering intestinal microbiome. NPJ Biofilms Microbiomes. (2025) 11:93. doi: 10.1038/s41522-025-00737-3 40461483 PMC12134233

[24] Wu H Jiang F Yuan W Zhao Y Liu N Miao X . Circadian time-dependent effects of experimental colitis on theophylline disposition and toxicity. Br J Pharmacol. (2024) 181:3743–59. doi: 10.1111/bph.16440 38862812

[25] Nahmias Y Androulakis IP . Circadian effects of drug responses. Annu Rev BioMed Eng. (2021) 23:203–24. doi: 10.1146/annurev-bioeng-082120-034725 33788580 PMC8525652

[26] Dunlap JC . Molecular bases for circadian clocks. Cell. (1999) 96:271–90. doi: 10.1016/S0092-8674(00)80566-8 9988221

[27] Butler TD Downton P Dickson SH Luengas-Martinez A Simpkins DA Khoo I . Colonic inflammation modulates the intestinal circadian landscape. iScience. (2025) 28:113183. doi: 10.1016/j.isci.2025.113183 40822350 PMC12355569

[28] De Mey C Meineke I . Prandial and diurnal effects on the absorption of orally administered enteric coated 5-aminosalicylic acid (5-ASA). Br J Clin Pharmacol. (1992) 33:179–82. doi: 10.1111/j.1365-2125.1992.tb04021.x 1550697 PMC1381304

[29] Smolensky MH Peppas NA . Chronobiology, drug-delivery, and chronotherapeutics. Adv Drug Delivery Rev. (2007) 59:823–4. doi: 10.1016/j.addr.2007.08.004 17884237

[30] Bórquez DA Urrutia PJ . Iron regulatory protein 1: The deadly switch of ferroptosis. Neural Regener Res. (2023) 19:477–8. doi: 10.4103/1673-5374.380889 37721261 PMC10581575

[31] Long D Mao C Huang Y Xu Y Zhu Y . Ferroptosis in ulcerative colitis: Potential mechanisms and promising therapeutic targets. BioMed Pharmacother. (2024) 175:116722. doi: 10.1016/j.biopha.2024.116722 38729051

[32] Wang X Quan J Xiu C Wang J Zhang J . Gegen qinlian decoction (GQD) inhibits ulcerative colitis by modulating ferroptosis-dependent pathway in mice and organoids. Chin Med. (2023) 18:110. doi: 10.1186/s13020-023-00819-4 37649073 PMC10466729

[33] Stockwell BR Friedmann Angeli JP Bayir H Bush AI Conrad M Dixon SJ . Ferroptosis: A regulated cell death nexus linking metabolism, redox biology, and disease. Cell. (2017) 171:273–85. doi: 10.1016/j.cell.2017.09.021 28985560 PMC5685180

[34] Li E Li X Huang J Xu C Liang Q Ren K . BMAL1 regulates mitochondrial fission and mitophagy through mitochondrial protein BNIP3 and is critical in the development of dilated cardiomyopathy. Protein Cell. (2020) 11:661–79. doi: 10.1007/s13238-020-00713-x 32277346 PMC7452999

