# Supplementary material for: Chronopharmacological effects of 5-aminosalicylic acid in the treatment of experimental colitis in mice
Source: Front Immunol. 2026 Oct 2;17:1936952. doi: 10.3389/fimmu.2026.1936952 (PMC13671935; doi:10.3389/fimmu.2026.1936952)
Supplement: Supplementary file 1 [file DataSheet1.pdf]

Figure S1

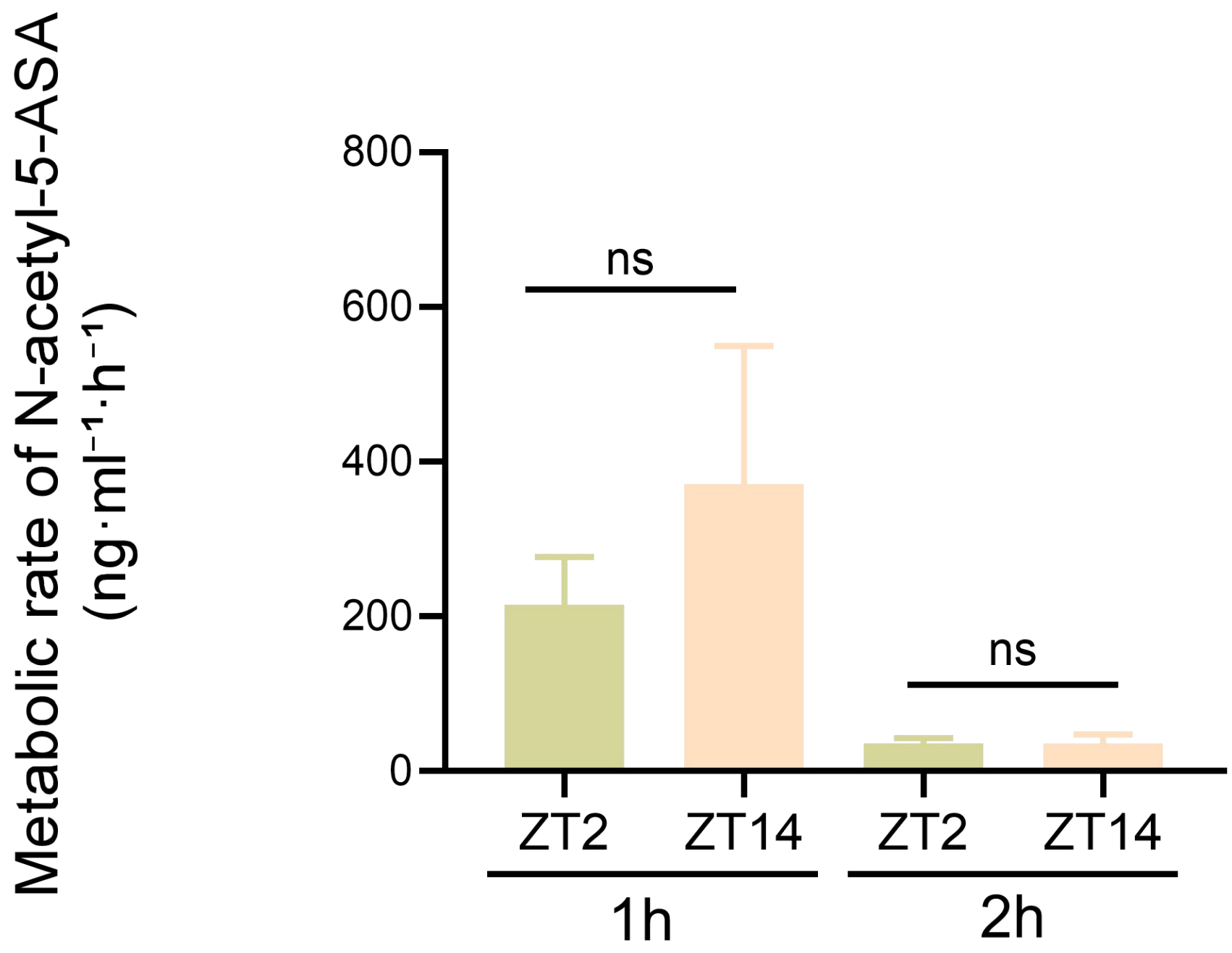

Figure S1. Formation rate of N-acetyl-5-ASA following 5-ASA administration at ZT2 and ZT14.

Figure S2

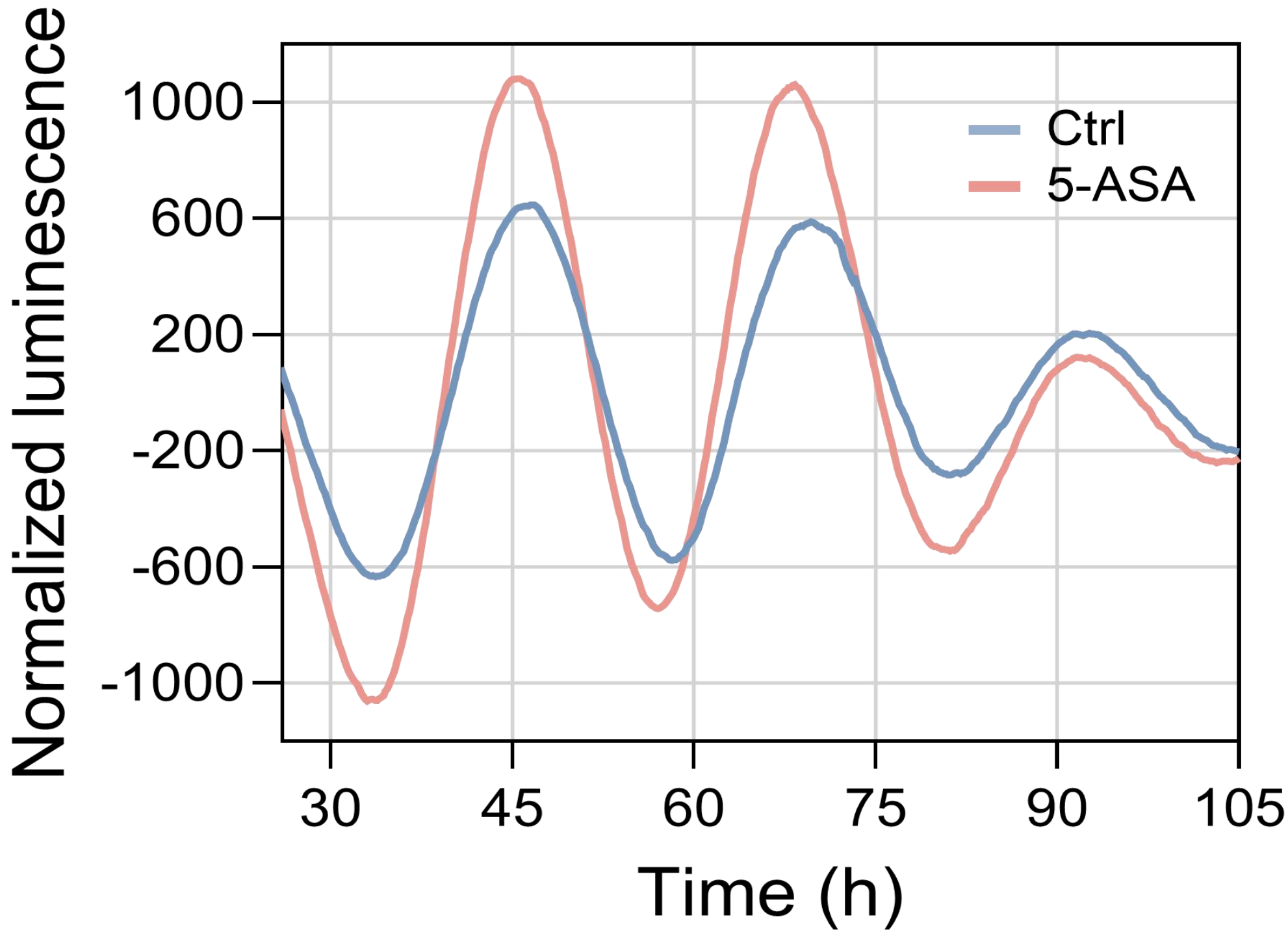

Figure S2.Normalized luminescence traces of *Bmal1*-luc showing circadian rhythmicity in 5-ASA-treated and control groups.
